# Host species-specific mutations in the thumb domain of the 3D^pol^ polymerase are required for efficient replication of human hepatitis A virus in mice

**DOI:** 10.1371/journal.ppat.1014213

**Published:** 2026-05-11

**Authors:** Ichiro Misumi, Takayoshi Shirasaki, Ling Xie, Bryan Yonish, Olga González-López, Asuka Hirai-Yuki, Xian Chen, You Li, Jason K. Whitmire, Stanley M. Lemon

**Affiliations:** 1 Department of Genetics, The University of North Carolina at Chapel Hill, Chapel Hill, North Carolina, United States of America; 2 Department of Microbiology & Immunology, The University of North Carolina at Chapel Hill, Chapel Hill, North Carolina, United States of America; 3 Department of Biochemistry and Biophysics, The University of North Carolina at Chapel Hill, Chapel Hill, North Carolina, United States of America; 4 Lineberger Comprehensive Cancer Center, The University of North Carolina at Chapel Hill, Chapel Hill, North Carolina, United States of America; 5 Department of Pediatrics, The University of North Carolina at Chapel Hill, Chapel Hill, North Carolina, United States of America; 6 Department of Medicine, The University of North Carolina at Chapel Hill, Chapel Hill, North Carolina, United States of America; Duke-NUS Medical School, SINGAPORE

## Abstract

Hepatitis A virus (HAV) is a globally important cause of enterically-transmitted hepatitis. It is one of 9 distinct *Hepatovirus* species in the *Picornaviridae,* among which phylogenetic reconstructions suggest multiple past host species jumps. HAV readily infects mice with defective type I interferon responses, suggesting the major barrier preventing human HAV from replicating in a rodent host is an inability to overcome innate immune responses. In prior studies, only a single nonsynonymous mutation of uncertain significance (3D^pol^-R468K) was identified within the genome of wild-type HAV following passage in interferon-receptor knockout mice. Here, we show that R468K and other mutations in the 3D^pol^ polymerase (E461D and D473G) are uniformly present in virus recovered from *Ifnar1*^*-/-*^ mice following intrahepatic injection of HAV RNA. Reverse molecular genetics experiments confirmed RNAs with R468K or D473G mutations were more likely to initiate sustained infection than wild-type RNA in mice. In competition experiments using cell culture-adapted virus, a K468 mutant out-replicated wild-type R468 in murine cells, whereas R468 rapidly replaced K468 in human cells. These 3D^pol^ mutations thus promote HAV replication in a host species-specific manner. AlphaFold 3 modeling indicates E461, R468, and D473 are closely positioned on the surface of the 3D^pol^ thumb domain, suggesting they modulate interactions with species-specific host factor(s). Proteomics analysis of proteins co-precipitating with HA-3D^pol^ expressed in Huh-7.5 cells identified heat shock 70 protein HSPA8 and its co-chaperone, BAG2. HSPA8 is known to be a critical hepatovirus host factor and HAV genome replication is highly dependent upon heat shock chaperone activity. The mouse-adaptive R468K mutation enhances co-immunoprecipitation of 3D^pol^ with murine HSPA8 and BAG2, suggesting it facilitates chaperone-dependent acquisition of polymerase function in mouse cells. Our results identify a non-immune barrier to HAV replication in mice and enable future reverse molecular genetics studies in a small animal model.

## Author summary

Given an ability to rapidly mutate and undergo genetic selection, viruses with RNA genomes pose unique risks of zoonotic spread from animal hosts to humans. Although viruses closely related to human hepatitis A virus (HAV) infect many other mammalian species and are known to have undergone multiple past shifts in host species, HAV was considered for many years to be capable of infecting only a limited number of primate species. However, our recent studies show HAV, unlike other human hepatitis viruses, replicates efficiently in genetically-modified mice lacking effective antiviral interferon responses. This suggests that an inability to overcome innate immune responses prevents the spread of HAV to other animal species. Here, we define a second, nonimmune barrier to HAV infection in mice. We show efficient replication in mice requires mutations altering the surface of its RNA-dependent RNA polymerase, 3D^pol^, the presumed catalytic core of its replication machinery. We find that the polymerase interacts with heat shock pathway chaperone proteins, and suggest that mouse-adaptive mutations promote host species-specific chaperone-dependent acquisition of polymerase function. Our data enhance understanding of the biology underlying this human pathogen, and make possible reverse molecular genetics studies of HAV in a readily accessible small animal model.

## Introduction

Both RNA and DNA viruses pose risks for zoonotic transmission leading to the emergence of new epidemic infectious diseases in human populations [1]. However, an array of genetic and immunologic as well as epidemiologic factors limit the potential for cross-species transmission of most viruses and thus act as barriers to shifts in viral host species. These barriers include host-species specific factors required for viral entry into cells or viral genome replication, and the need to disrupt or evade species-specific restriction factors and innate immune antiviral responses [2–4]. The human viruses responsible for type B hepatitis *(Hepadnavirus*) and type C hepatitis (*Hepacivirus*) are both tightly restricted in their host range, and are capable of robustly infecting only humans or other closely-related nonhuman primate species [5,6]. This strict host species tropism prevails even in animals deficient in innate and adaptive antiviral immune responses, and has confounded efforts to develop small animal models for these two hepatotropic viruses. By contrast, hepatitis A virus (HAV), a picornavirus classified in the genus *Hepatovirus*, replicates efficiently in *Ifnar1*^*-/-*^ mice that do not express the type I interferon receptor, and in *Mavs*^*-/-*^ and *Irf3*^*-/-*^*Irf7*^*-/-*^ mice that lack effective type I interferon responses [7]. *Ifnar1*^*-/-*^ mice provide a useful model of human hepatitis A, facilitating studies of immune responses and disease pathogenesis and the evaluation of candidate antiviral therapies [8–11].

The robust replication of human HAV in mice with impaired type I interferon responses suggests that the major factor restricting the ability of HAV to infect immunocompetent adult mice is its inability to evade a punishing antiviral type I interferon response induced by early rounds of replication in the liver [4,7]. Indeed, a broad-based intrahepatic interferon-stimulated gene (ISG) response occurs in normal B6 mice 12–18 hours after intravenous inoculation of the virus and has been linked to abortive replication of the viral genome [4]. Although how HAV normally evades innate immune responses in human cells is poorly understood, previous studies suggest that proteolytically-active nonstructural proteins expressed by the virus may degrade key signaling molecules involved in the induction of an antiviral response, including mitochondrial antiviral-signaling protein (MAVS), TIR domain-containing adapter molecule 1 (TRIF), and inhibitor of nuclear factor kappa B kinase regulatory subunit gamma (IKBKG, *a.k.a.* NEMO) [12–16]. These and likely other HAV adaptations are specific for human proteins and are unable to disrupt analogous antiviral responses induced in mice [4].

In our initial studies of HAV infection in mice, virus present in feces from an experimentally-infected chimpanzee was passaged in *Ifnar1*^*-/-*^*Ifngr1*^*-/-*^ (DKO) mice that are doubly-deficient for receptors of both type I and type II interferons [7]. Robust replication followed a delay of several weeks after intravenous inoculation of the virus. Infection was associated with substantial hepatic inflammation, leading to significant increases in serum levels of alanine aminotransferase (ALT), a standard marker of hepatocellular injury [7]. As in human infections, virus was shed in feces, thereby allowing successive rounds of cell-free virus passage in naïve animals [7,17]. Sequencing of the nearly complete viral genome from mice at the second and fourth mouse passage levels identified only a single non-synonymous mutation in the viral genome. The conservative nature of this mutation, an Arg to Lys substitution at residue 468 of the 3D^pol^ RNA-dependent RNA polymerase [7], suggested little need for genetic adaptation of the virus in mice, but the significance of this R468K mutation was not assessed.

Here, we describe a series of studies showing that 3D^pol^-R468K, and other mutations we identify within 3D^pol^ in viruses recovered from mice after direct intrahepatic injection of wild-type viral RNA, are crucially important for replication of human HAV in the murine liver. HAV is unique among the picornaviruses in that the replication of its RNA genome is dependent upon the heat shock chaperone HSP70/HSP90 pathway [11]. We show here that 3D^pol^, when expressed in a human hepatocyte-derived cell line, forms a complex with the 70kd heat shock chaperone protein HSPA8, a well-documented host factor required for HAV replication [11,18], and its specific regulatory partner, BAG2 [19,20]. We provide evidence that the R468K mutation in 3D^pol^ facilitates its interactions with the murine orthologs of HSPA8 and BAG2, suggesting that the mouse adaptive mutations in 3D^pol^ promote heat shock chaperone-dependent acquisition of polymerase function in a host species-specific manner.

## Results

### Genetic changes associated with HAV passage in *Mavs*^*-/-*^ mice

**Fig 1:**
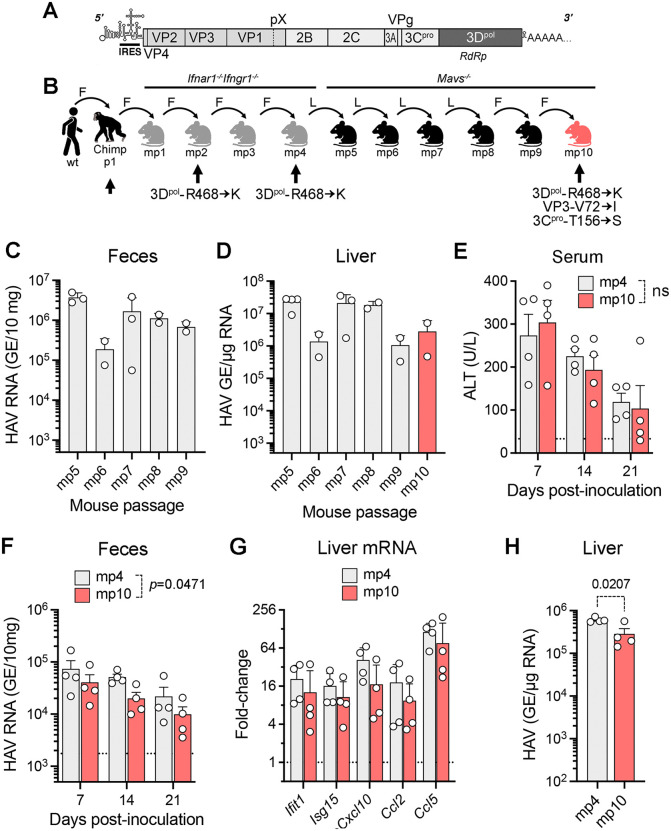
Murine passage of HM175 virus. (A) Organization of the HAV RNA genome. The polyprotein-coding region is shown as a box with the 3D^pol^ RNA-dependent RNA polymerase (RdRp) deeply shaded. IRES, internal ribosome entry site. (B) Virus passage in DKO and *Mavs*^*-/-*^ mice following its recovery from a chimpanzee infected with human fecal material. Arrows indicate sequencing of the viral genome, with amino acid substitutions from chimp-p1 virus listed below. Passage from chimp-p1 to mp4 virus has been reported previously [7]. Subsequent passage from mp5 to mp10 was in male *Mavs*^*-/*-^ mice. wt, wild-type; mp, mouse passage; F, fecal inoculum; L, liver inoculum. (Created in part in BioRender: Lemon, S. (2026) https://BioRender.com/7oozx5a.) (C) Fecal HAV RNA shedding 14 days postinfection and (D) viral load in liver at necropsy during successive mouse passages. n = 2-4 male mice at each passage. (E) Serum alanine aminotransferase (ALT), (F) fecal HAV RNA shedding, (G) increases from baseline in intrahepatic cytokine transcript levels, and (H) viral load in liver at necropsy 21 days following i.v. inoculation of 2.5 x10^5^ GE mp4 or mp10 virus in *Ifnar1*^*-/-*^ mice. n = 4 male mice in each cohort; p-values by two-way ANOVA corrected for multiple comparisons by the two-stage step-up method of Benjamini, Krieger and Yeketueli (panels E-G) or two-sided t-test (H). ns = nonsignificant. p-values for individual cytokine transcript increases in mp4- versus mp10-infected mice were >0.05.

In previous studies, we inoculated *Ifnar1*^*-/-*^*Ifngr1*^*-/-*^ DKO mice with a fecal extract from a chimpanzee that was experimentally-infected with human HAV (‘chimp-p1’ virus) [7,21] (Fig 1A and 1B). Viral replication was evident in the DKO mice by 14 days post-inoculation (dpi), with serum ALT and fecal virus shedding both peaking 35 dpi [7]. Virus shed in feces was passaged subsequently four times in DKO mice (Fig 1B), with the course of the infection accelerating and peak viral replication occurring by 7 dpi [7]. Surprisingly, even after 4 passages in mice (‘mp4’ virus), the only change in the viral polyprotein was the conservative 3D^pol^-R468K mutation caused by an adenine to guanine substitution at nucleotide 7351 [7] (Fig 1B). These early, previously published studies thus suggested minimal if any requirement for genetic adaptation of HAV during the shift from a primate to an immunologically-impaired rodent host [7].

To determine whether further passage of the virus in mice would lead to additional genetic changes, we subjected the mp4 virus to six additional serial passages in adult male *Mavs*^*-/-*^ mice that are deficient in MAVS and thus lack any interferon response to the virus [7] (Fig 1B). *Mavs*^*-/-*^ mice are equally if not more permissive to HAV replication than DKO or *Ifnar1*^*-/-*^ mice, and unlike *Ifnar1*^*-/-*^ mice show neither detectable innate immune responses nor evidence of liver injury due to a lack of interferon regulatory factor 3 (IRF3) activation [7,8]. Male mice were used exclusively as previous studies show higher levels of replication in male versus female mice [22]. Four serial passages of mp4 virus (mp5 to mp8) were carried out by intravenous inoculation of 20% liver extracts, each collected 14 dpi, with two additional passages (mp9 and mp10) initiated by intravenous inoculation of fecal extracts (Fig 1B). Virus is present in both liver and feces of infected animals, and it makes little difference which source is used to passage the virus [7]. However, passage of fecal virus could be considered a more definitive test of the virus having replicated through all stages of the life cycle, including viral egress through the biliary tract, and was thus used for the final two viral passages in *Mavs*^*-/-*^ mice. Despite considerable variation in individual animals, there were no significant trends over these 6 passages in either fecal virus shedding or viral load in the liver 14 dpi (Fig 1C and 1D). By the 10^th^ passage, the virus had been replicating for 213 days in mice: 125 days in DKO mice (mp1 to mp4), and 88 days in *Mavs*^*-/-*^ mice (mp5 to mp10). The nearly complete sequence of mp10 virus (nucleotides 24–7478) was determined by automated Sanger sequencing of RT-PCR amplimers as carried out previously for the mp2 and mp4 virus [7]. Compared with mp4 virus [7], there were 9 nucleotide substitutions in mp10, all but one transition mutations, and only two of which were nonsynonymous: G1683→A (VP3-V72I), and C5758→G (3C^pro^-T156S) (S1 Table). Interestingly, both G1683 and C5758 were 100% conserved among 100 *Hepatovirus A* sequences downloaded from GenBank, although 3C^pro^-156 is Ala in 10% of these due to a polymorphism at nucleotide 5757. Compared with the chimp-p1 virus inoculum used to initiate passage in mice [7], there were only 7 nucleotide substitutions and 3 amino acid changes (VP3-V72I, 3C^pro^-T156S, and 3D^pol^-R468K) in the mp10 polyprotein (Fig 1B).

To determine whether the additional genetic changes accumulating in the virus during extended passage in *Mavs*^*-/-*^ mice alter its replication capacity or pathogenicity, male *Ifnar1*^*-/*-^ mice were inoculated intravenously with liver extracts containing equivalent amounts (2 x10^5^ genome equivalents, GE) of mp4 or mp10 virus. Although the two inocula induced comparable elevations of serum ALT activity (Fig 1E), fecal viral shedding, intrahepatic ISG transcript abundance, and liver viral load at 21 dpi were all modestly reduced in mp10 versus mp4-infected mice (Fig 1F-1H). These results suggest the single conservative amino acid substitution found in mp2 virus, 3D^pol^-R468K, is sufficient for optimal replication in mice, but they leave unanswered the question as to whether any genetic adaptation is required for efficient replication.

### Rescue of infectious HAV following intrahepatic injection of wild-type RNA in mice

**Fig 2:**
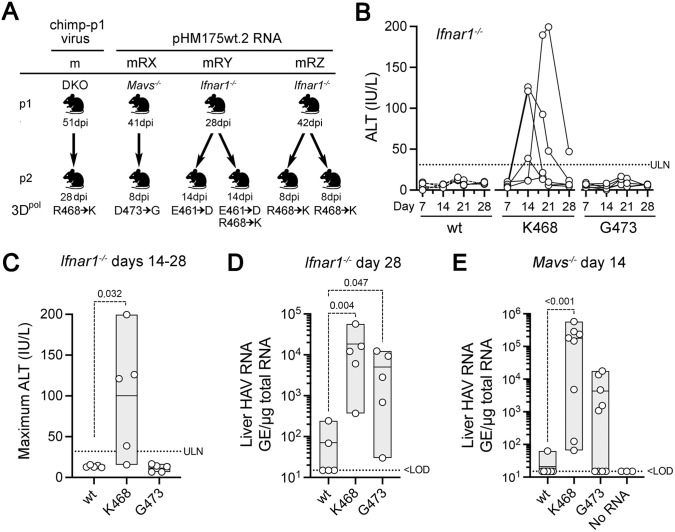
HAV infection of *Ifnar1*^*-/-*^ or *Mavs*^*-/-*^ mice following intrahepatic injection of *in vitro*-transcribed HAV RNA. (A) Amino acid substitutions in 3D^pol^ in 3 independent murine lineages of HAV established by intrahepatic inoculation of *in vitro* transcribed wild-type HAV RNA (mRX, mRY, and mRZ). Also shown is the previously reported m lineage established by i.v. inoculation of chimp-p1 virus [7]. Each symbol reflects a single infected mouse. All second passage (p2) mice were *Ifnar1*^*-/-*^ except for the m lineage, which was DKO. For additional details, see Table 1. dpi = days post-inoculation at necropsy. (Created in part in BioRender: Lemon, S. (2026) https://BioRender.com/7oozx5a.) (B) Temporal pattern of serum alanine aminotransferase (ALT) elevations, and (C) maximum ALT elevation, in male *Ifnar1*^*-/-*^ mice inoculated directly with 10μg wild-type (wt, 3D^pol^-R468), 3D^pol^-K468, or 3D^pol^-G473 RNA by intrahepatic injection. n = 5 mice in each cohort; bars represent range, line = mean. p-value by ANOVA with Dunn’s correction for multiple comparisons. (D) Intrahepatic viral RNA abundance 28 days after inoculation of RNA as in panels B. GE: genome equivalents. n = 5 mice in each cohort; p-value by ANOVA with Dunn’s correction for multiple comparisons. (E) Intrahepatic HAV RNA in male *Mavs*^*-/-*^ mice 14 dpi intrahepatic injection of the indicated viral RNA. n = 8-9 mice in each cohort (n = 3 for no RNA); p-value by ANOVA with Dunn’s correction for multiple comparisons.

To determine whether any adaptive mutation is necessary for efficient replication in mice, we challenged mice by direct intrahepatic injection of RNA transcribed from pHAVwt.2, a plasmid containing wild-type HM175 virus sequence [23]. The sequence of this infectious molecular clone differs from the chimp-p1 sequence (GenBank KX343014.1) at only 3 base positions, none of which alter the amino acid sequence of the polyprotein (S1 Table). In previous studies, intrahepatic injection of plasmid-derived HAV RNA was shown to be an efficient method for infecting nonhuman primates [24,25]. In exploratory experiments, a total of 17 mice with compromised type 1 interferon responses, including 11 *Mavs*^*-/-*^ (7 males and 4 females) and 6 *Ifnar1*^*-/-*^ (all males) mice, were inoculated with 0.1-50 μg genome-length wild-type HAV RNA by direct intrahepatic injection in 3 independent experiments (Table 1). The animals were monitored periodically for ALT elevation or fecal virus shedding and liver tissue assayed for viral RNA at necropsy. Only three of these mice, all male, 2 *Mavs*^*-/-*^ and 1 *Ifnar1*^*-/-*^, each injected with ≧10 μg RNA, developed evidence of HAV infection after intervals ranging from 28 to 42 days. Virus present in liver of each of these 3 animals at necropsy was successfully passaged to naïve *Ifnar1*^*-/-*^ mice (2^nd^ mouse passage), in each case inducing substantial serum ALT elevations by 7 dpi and thereby establishing 3 new, independent murine viral lineages derived from transcribed viral RNA: mRX, mRY, and mRZ (Fig 2A and Table 1). The nearly complete nucleotide sequence (nts 41–7467) of the second passage mRX virus (‘mRXp2’) was determined by Sanger sequencing of RT-PCR amplified cDNA. The only change identified from wild-type HAV was an A to G substitution at nucleotide 7366 (7366A→G) resulting in an Asp to Gly substitution at residue 473 of the polymerase (3D^pol^-D473G) (Fig 2A and Table 1). The same mutation was present in mRXp1 virus recovered from the original RNA-inoculated *Mavs*^*-/-*^ mouse. Remarkably, this mutation is only 5 residues distant from the 3D^pol^-R468K mutation identified in chimp-p1 virus following its first two passages in mice (Fig 1B) [7].

**Table 1:** Virus recovered from mice following intrahepatic inoculation of wild-type HAV RNA.

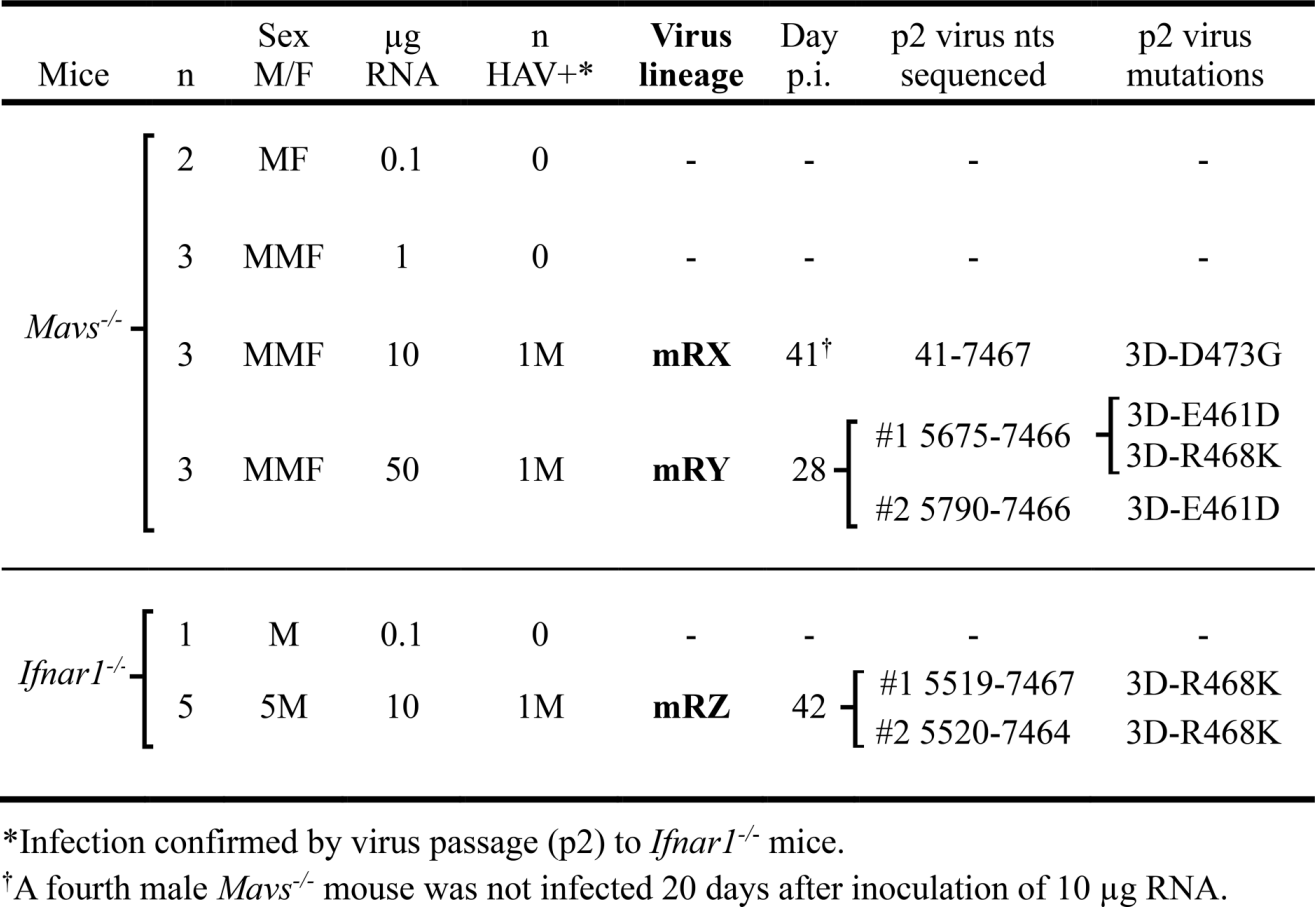

Given that the only mutations identified in the mouse-adapted chimp-p1 (mp2 and mp4) virus (Fig 1B) [7] and in the mRXp2 viral polyprotein were in the polymerase, we limited sequencing of the mRYp2 and mRZp2 viruses to 3D^pol^ and surrounding sequence (nts 5790–7467). A G7331→U mutation (3D^pol^-E461D) was identified in mRYp2 virus recovered from the livers of each of two *Ifnar1*^*-/-*^ mice following the second mouse passage, while the G7351→A mutation (3D^pol^-R468K) was additionally present in virus from one of these mice (Fig 2A and Table 1). Remarkably, the 3D^pol^-R468K mutation was also identified in mRZp2 virus from two *Ifnar1*^*-/-*^ mice (Fig 2A and Table 1). Thus, each of the 3 murine viral lineages arising from intrahepatic injection of in vitro-transcribed wild-type viral RNA had sustained, nonsynonymous mutations at E461, R468, or D473 of 3D^pol^, near the carboxy terminus of the polymerase. We confirmed the plasmid-related origin of each of the 3 new murine viral lineages by demonstrating the presence of synonymous nucleotide differences existing between the wild-type pHAVwt.2 plasmid and chimp-p1 virus [7] at nucleotides 4185, 6216, and 6522 (S1 Table).

### Carboxy-terminal 3D^pol^ mutations promote viral rescue from RNA in mice

To determine whether 3D^pol^-R468K and 3D^pol^-D473G are truly mouse-adaptive mutations, they were introduced into the pHAV-wt.2 plasmid, and 10 μg RNA transcribed from the modified plasmids was delivered by direct intrahepatic injection into groups of male *Ifnar1*^*-/-*^ and *Mavs*^*-/-*^ mice. Serum ALT activities became elevated in 4 of 5 *Ifnar1*^*-/-*^ mice receiving 3D^pol^-K468 RNA (in 3 of 4 mice by 14 dpi), whereas no significant ALT elevations occurred in mice injected with 3D^pol^-G473 or wild-type RNA over 28 days observation (Fig 2B and 2C). Nonetheless, compared with *Ifnar1*^*-/-*^ mice injected with wild-type RNA, the intrahepatic viral load was significantly increased in livers of mice injected with 3D^pol^-G473 as well as 3D^pol^-K468 RNA at 28 dpi (Fig 2D). Similar increases (>10- to 100-fold above wild-type RNA) were observed at 14 dpi in 7 of 8 *Mavs*^*-/-*^ mice injected with 3D^pol^-K468 RNA, and 5 of 9 *Mavs*^*-/-*^ mice injected with 3D^pol^-G473 RNA (Fig 2E). Collectively, these results show that the 3D^pol^-R468K and 3D^pol^-D473G mutations enhance the capacity of virus to infect and replicate in mice.

### Replication fitness of 3D^pol^ mutant virus in human and murine cell cultures

**Fig 3:**
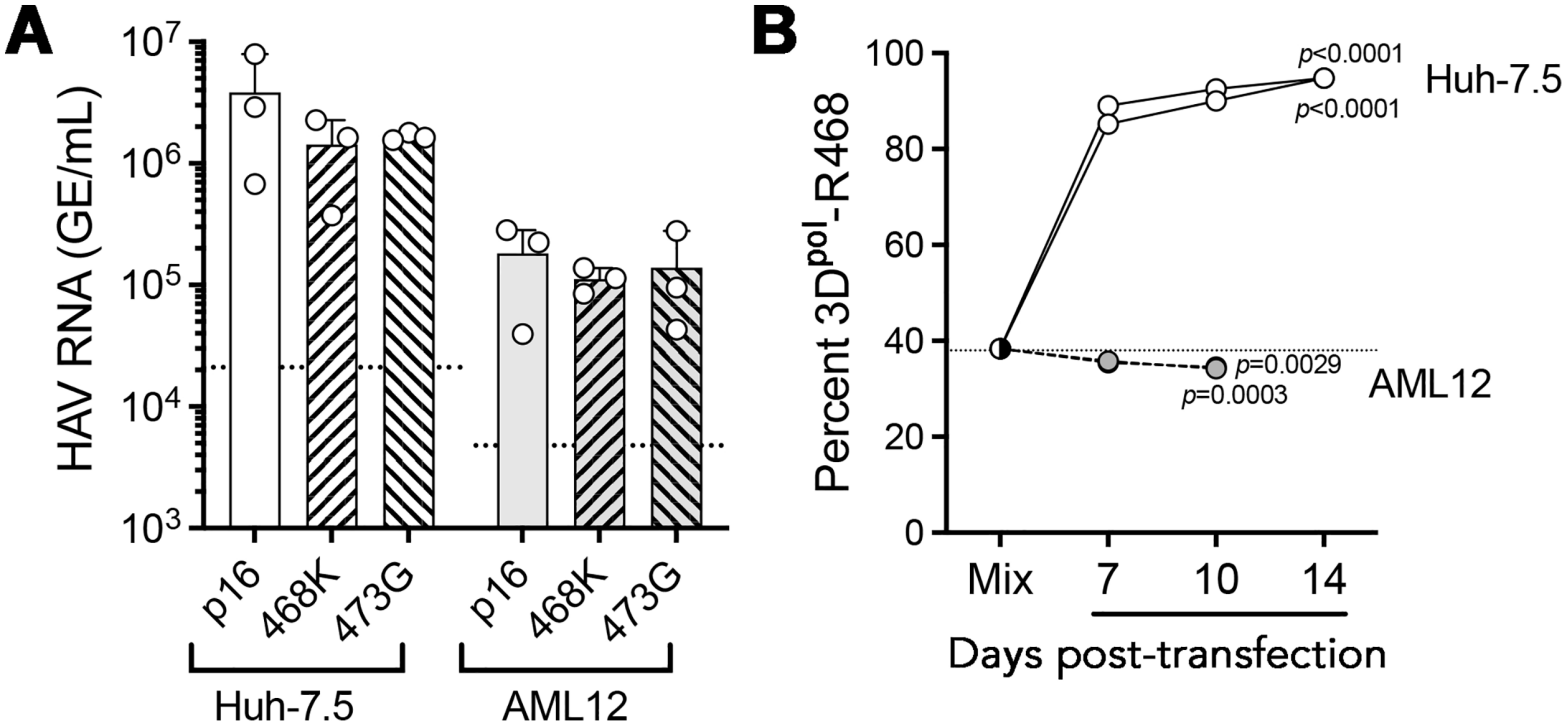
Species-specific replication fitness conferred by 3D^pol^-468 alleles in cell culture. (A) HAV RNA abundance in lysates of human (Huh-7.5) versus murine (AML12) hepatocytes 5-7 days following transfection of *in vitro* transcribed p16 (3D^pol^-R468), p16/3D^pol^-K468, or p16/3D^pol^-G473 RNA. Data are mean values from 3 independent experiments, each with 2-3 technical replicates. The dashed lines indicate mean HAV RNA abundance in each cell type (n = 3) at harvest when treated with the potent antiviral RG7834 (200nM) [29] immediately after transfection. (B) Percentage of HAV transcripts with the 3D^pol^-R468 allele determined by high-throughput RNA sequencing of RT-PCR amplimers from cultures of Huh-7.5 and AML12 cells (two independent cultures of each) following transfection of a nominal 50:50 mixture of p16 (3D^pol^-R468) and p16/3D^pol^-K468 RNA. Read counts ranged from 7820 in the inoculum mix, to a mean of 7817 at day 14 in Huh-7.5 cells and 620 at 10 days in AML12 cells. HAV RNA abundance was insufficient for quantitation at day 14 in AML12 cells. p-values by Fisher’s exact test; p = 0.009 for cell type-specific differences between days 0-10 by two-way repeated measures ANOVA.

Because wild-type HAV replicates poorly in cell culture [26,27], we separately introduced the 3D^pol^-R468K and 3D^pol^D473G mutations into an infectious molecular clone of the cell culture-adapted HM175/p16 virus [28]. Genome-length RNAs transcribed from these clones were then transfected into Huh-7.5 cells, which are human in origin, and AML12 cells, which are transformed murine hepatocytes. All three RNAs (p16, 3D^pol^-K468, and 3D^pol^-G473) were replication competent, as evidenced by large differences in the abundance of viral RNA compared with cells treated with a potent antiviral compound (RG7834) [29] following transfection: log_10_ fold-difference 2.3-2.6 in Huh-7.5 cells, and 1.7-2.5 in AML12 cells (Fig 3A). The abundance of the mutant 3D^pol^-K468 and 3D^pol^-G473 p16 RNAs was similar to that of the parental p16 virus (3D^pol^-R468) in both cell types 7 days after transfection (Fig 3A). However, the abundance of both mutants as well as the p16 parent was 10-fold less in the mouse cells, suggesting less efficient replication.

To better assess the impact of the 3D^pol^R468K mutation on replication fitness, we transfected human and murine cells (two replicate cultures of each) with an approximately equal mixture of the p16 (3D^pol^-R468) and p16/3D^pol^-K468 RNAs. RNA was extracted from the cells between 7 and 14 days post-transfection, and RT-PCR amplimers derived from the 3D^pol^ coding region subjected to high-throughput sequencing to determine the relative abundance of 3D^pol^-R468 versus 3D^pol^-K468 reads. In Huh-7.5 cells, the R468 allele rapidly became dominant, increasing from 38.3% in the transfected RNA inoculum mix to a mean of 87.1% (range 85.2-89.1%) by 7 days post-transfection, and 94.8% range (94.8-94.8% range) by day 14 (p < 0.0001 by Fisher’s exact test) (Fig 3B). In sharp contrast to Huh-7.5 cells, in which the overall HAV RNA abundance increased following an initial dip after transfection, the abundance of HAV RNA dropped >75-fold in AML12 cells between days 7 and 10 post-transfection and was no longer quantifiable at day 14. This likely reflects a much more active innate immune response to the transfected RNA in AML12 cells, that demonstrate a robust innate immune response to RNA virus infection in contrast to Huh-7.5 cells that are defective in retinoic acid-inducible gene I (RIG-I) sensing of double-stranded RNA [30,31]. Alternatively, there may be restrictions to replication of HAV in the murine cells beyond those forcing selection of the mutations we have identified in 3D^pol^. Nonetheless, the percentage of R468 reads in the AML12 cells dropped from 38.3% to a mean of 35.6% at 7 days and 34.4% at 10 days (p < 0.0029), with little difference between the two cultures transfected with the RNA mixture. This suggests that the 3D^pol-^K468 virus has a small but measurable survival advantage in the murine cells. Collectively, these data show the host species-specific nature of replication fitness associated with the 3D^pol^-R468 versus 3D^pol^-K468 alleles.

### The 3D^pol^R468K mutation does not rescue replication of cell culture-adapted p16 virus in mice

**Fig 4:**
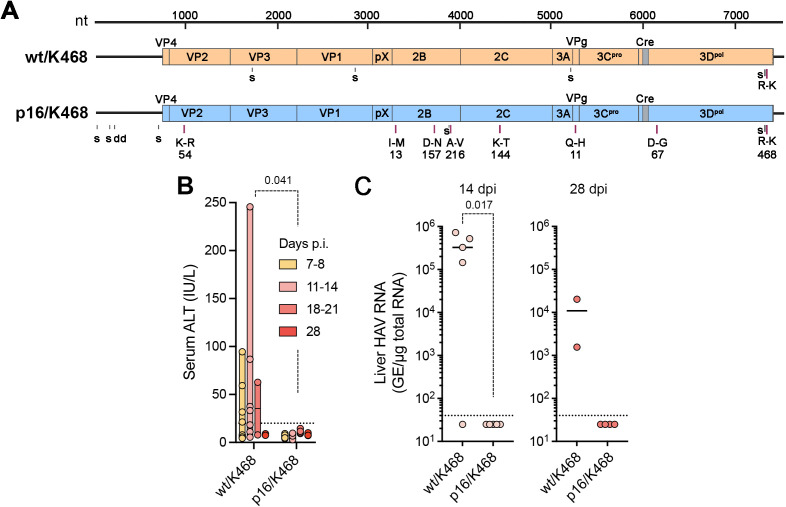
Murine attenuation phenotype of cell culture-adapted p16 virus. (A) Genome-length infectious molecular clones of wild-type (wt) HM175 strain HAV and related cell-culture adapted p16 virus, each engineered to contain the mouse-adaptive 3D^pol^-R468K mutation. Differences from the sequence of chimp.p1 virus used to initiate HAV infection in mice (Fig 1A) [7] are indicated: ‘s’, nucleotide substitution (silent if in open reading frame); ‘d’, nucleotide deletion; ‘dup’, duplication, ‘K-R’, Lys to Arg substitution; ‘cre’, *cis*-acting RNA replication element. (B) Serum ALT activities in male *Ifnar1*^*-/-*^ mice 7-28 days after direct intra-hepatic inoculation of 50 μg RNA transcribed from plasmids containing the genomes shown in panel A. n = 9 mice inoculated with p16/K468 and 7 with wt/K468 RNA, with 5 of each sacrificed at 14 days post-inoculation (dpi) and the remainder at 28 dpi; p-value by mixed effect analysis with Geisser-Greenhouse correction. Dashed line = upper limit of normal. (C) Intrahepatic HAV RNA abundance 14 (n = 5) and 28 (n = 2-5) dpi of RNAs as in panel (B). p-value (14 dpi) by two-tailed Welch’s t-test. Dashed line = limit of detection.

Wild-type HAV replicates very inefficiently in cell culture, but will adapt with continued passage leading to a more rapid replication cycle, greater virus yields, and even visible cytopathic effects [27,28,32]. HAV strains that are well adapted to growth in cell culture are typically attenuated for both replication and disease pathogenesis in nonhuman primates, a phenotype linked primarily to mutations in the viral 2B and 2C proteins [33,34]. Consistent with these early studies, Li et al. [35] reported recently that the RNA genome of our mouse-passaged mp4 virus (which contains the 3D^pol^-R468K mutation) (Fig 1A) failed to generate infectious virus when transfected into Huh-7.5.1 cells. Replication could be rescued in cell culture, however, by the introduction of two previously reported cell-culture adaptive mutations (2B-A216V and 2C-F76S) [35]. This new recombinant virus, named “HAV-2m”, replicated well and induced hepatitis when inoculated intravenously into *Ifnar1*^*-/-*^ mice, thus providing a useful link between cell culture infectivity and the capacity to infect mice. This recent report prompted us to ask whether introducing the 3D^pol^-R468K mutation into p16 would confer on it the capacity to infect mice. The cell culture-adapted p16 virus has 6 amino acid substitutions from wild-type virus including the 2B-A216V mutation inserted into HAV-2m, but it lacks the 2C-F76S mutation (Fig 4A). Previous studies showed it is highly attenuated in nonhuman primates [36]. We thus compared infectious outcomes in male *Ifnar1*^*-/-*^ mice following intrahepatic inoculation of 50μg p16/3D^pol^-K468 or wt/3D^pol^-K468 RNA. Over a 4 week follow-up period, ALT elevations were noted in 6 of 7 mice inoculated with the wt/3D^pol^-K468 RNA, but none of 9 mice inoculated with p16/3D^pol^-K468 RNA (Fig 4B). Furthermore, intrahepatic viral RNA abundance was > 10^5^ GE/μg total RNA in 4 of 5 mice 14 days after inoculation with the wildtype construct, but below the level of quantitation in each of the p16/3D^pol^-K468 inoculated animals (Fig 4C). We conclude that the mouse-adaptive 3D^pol^-R468K mutation is unable to rescue replication of the attenuated p16 virus in mice.

### Potential impact of 3D^pol^ mutations on RNA genome and polymerase structure

The mouse adaptive 3D^pol^ mutations identified in virus recovered from RNA-inoculated mice could promote replication either by altering essential RNA structure near the 3’end of the genome, or by changing the conformation of the polymerase, such that the viral RNA, or viral polymerase, are capable of more efficiently interacting with the murine ortholog of an important human hepatovirus host factor. The structure of the RNA genome has not been studied in detail and is not defined in this region of the genome. Thermodynamic modeling of the potential RNA structure, paired with a limited co-variant nucleotide substitution analysis, suggests the mouse adaptive mutations are located within two complex stem-loops (stem-loops iii and iv, numbered from the 3’ end of the genome) in which base pairing within the apical helices is predicted with high confidence (S1 Fig). Both structures are supported by the existence of co-variant substitutions distinguishing genotype V from genotype I *Hepatovirus A* viruses (highlighted in red boxes in S1 Fig). The nucleotide substitutions associated with murine adaptation have little overall impact on the predicted structure, preserving predicted base-pairing in each case except for the G→A change at nucleotide 7331 (E461D) which is located at the end of a short helix. This analysis, limited as it is, provides no evidence that the adaptive mutations operate at the level of RNA structure.

**Fig 5:**
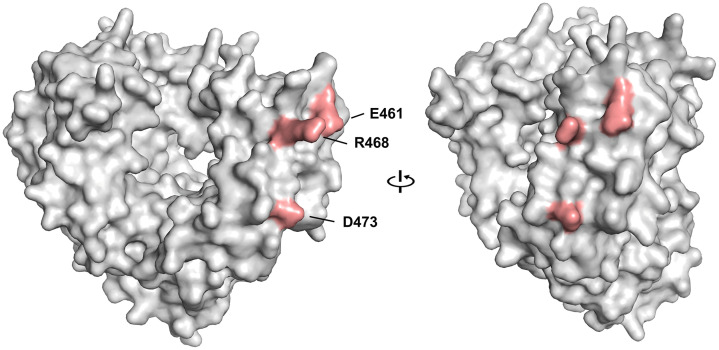
AlphaFold 3.0 prediction of the HAV 3D^pol^ polymerase structure. Residues involved in mouse-adaptive mutations (E461, R468, and D473) are highlighted in pink. The structure is predicted with very high confidence (path = 0.96) (see also S2 Fig).

By contrast, the position of the mutated amino acid residues within the AlphaFold 3 [37] predicted structure of the 3D^pol^ polymerase (Fig 5) suggests the adaptive mutations operate at the protein level, and likely enhance interactions with an unknown 3D^pol^ binding partner. Despite extensive previous efforts, the HAV polymerase has never been expressed in a catalytically active state [38]. To our knowledge, it has never been studied structurally. Nonetheless, a high confidence AlphaFold 3 prediction of its structure suggests close structural homology with the poliovirus 3D^pol^ polymerase (S2A and S2B Fig). The 3 residues involved in murine adaptation are solvent exposed on the surface of the predicted structure, and closely positioned within the thumb domain with the side chains of each facing outward from the predicted 3D^pol^ surface (Figs 5 and S2C-F). The adaptive mutations have negligible impact on the overall predicted structure of the polymerase (S2C-F Fig). The amino acid substitutions have no consistent effect on surface charge, but each ablates or shortens the length of the protruding side chains and, in the case of R468K, alters its predicted orientation (S2D-F Fig). A reasonable hypothesis to be drawn from these predictions is that the mouse adaptive mutations in 3D^pol^ alter the surface structure of the thumb domain to better accommodate the murine ortholog of a previously unrecognized human host factor that binds to the polymerase and enhances its capacity to support RNA synthesis.

### Candidate host 3D^pol^ interacting proteins

**Fig 6:**
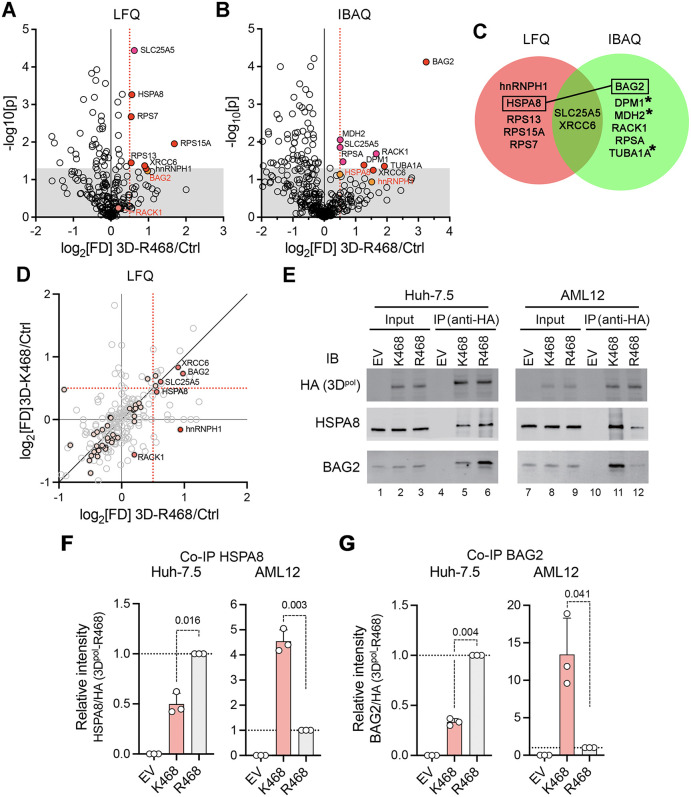
Candidate host 3D^pol^-interacting proteins. (A) Volcano plots showing fold-difference (FD) in protein abundance in anti-HA immunoprecipitates from lysates of cells expressing HA-tagged 3D^pol^-R468 versus control (Ctrl) cells transfected with an empty expression vector (n = 3 each) determined by label free quantitation (LFQ), plotted against significance. Heavily shaded symbols, log2[FD]≧0.5 and p < 0.05. (B) Similar volcano plot of FD determined by intensity based absolute quantitation (IBAQ) of protein abundance. See S4A and S4B Fig for similar volcano plots of proteins identified in HA-3D^pol^-K468 precipitates. (C) Venn diagram of proteins found to be significantly increased in abundance in precipitates from cells expressing HA-tagged 3D^pol^-R468 (log_2_[FD]≧0.5, p < 0.05) by either LFQ or IBAQ protocols. The functional linkage between HSPA8 and BAG2 is highlighted (see text). *Not quantifiable by LFQ analysis. (D) Fold-difference (from control cells) in LFQ-calculated protein abundance in immunoprecipitates from 3D^pol^-R468 cells, plotted against FD in immunoprecipitates from cells expressing HA-tagged 3D^pol^-K468. Lightly shaded symbols, p < 0.05 for 3D^pol^-R468 immunoprecipitates; heavily shaded symbols, proteins with log_2_[FD]≧0.5 and p < 0.05 by either LFQ or IBAQ protocols; dashed red line, p < 0.05. (E) Co-immunoprecipitation of HSPA8 and BAG2 with HA-3D^pol^-R468 or HA-3D^pol^-K468 expressed in human Huh-7.5 cells or murine AML12 cells. IP, immunoprecipitation; IB, immunoblot, with indicated antibodies. EV = empty vector. Data shown are from one of 3 co-immunoprecipitation experiments. (F,G) Quantitation of (F) HSPA8 and (G) BAG2 co-immunoprecipitation with HA-tagged 3D^pol^-R468 or 3D^pol^-K468 based on immunoblot intensities in n = 3 independent cxperiments carried out as in panel E. Blot intensities were normalized to the ratio of (F) HSPA8 or (F) BAG2 immunoprecipitated with HA-3D^pol^-R468 for each cell type. p-values by one-sample t-test.

To identify host proteins that may interact with 3D^pol^ and promote replication of the virus in a species-specific manner, we transiently expressed full-length HA-tagged 3D^pol^-R468 and 3D^pol^-K468 proteins in Huh-7.5 cells. Both proteins expressed only at very low levels, but distinct protein bands were readily visualized in immunoblots of anti-HA precipitates (S3A Fig). Triplicate sets of immunoprecipitates were subjected to liquid chromatography mass spectrometry (LS-MS) analysis (S3B Fig). A total of 391 host proteins were identified in in-gel digests of the immunoprecipitates, of which 357 were sufficiently abundant for intensity-based absolute quantification (IBAQ, S2 Table) and 233 for label free quantification (LFQ, S3 Table) (Fig 6A and 6B). Only a small number of these proteins were enriched in precipitates from cells expressing 3D^pol^-R468 versus precipitates from control cells transfected with empty vector using either method of analysis (Fig 6C). We thus adopted a low threshold for enrichment, log_2_ fold-difference (FD) ≧0.5 and p < 0.05, in order to avoid missing potential 3D^pol^ interactors. Thirteen proteins were found to be enriched in 3D^pol^-R468 precipitates by these low stringency criteria, including the heat shock protein 70 (HSP70) family member HSPA8 (heat shock protein family A member 8), and its co-chaperone, BAG2 (BAG family molecular chaperone regulator 2). Notably, HSPA8 is a well-documented host factor required for replication of HAV [11,18]. BAG2 has not been implicated previously in HAV replication, but it is known to interact directly with HSPA8 and to regulate its disengagement from client proteins [19,20]. HSPA8 and BAG2 were both identified in precipitates of the mouse-adapted 3D^pol^-K468 protein expressed in the human cells, but only BAG2 reached significance (by IBAQ analysis) (Figs 6D, S4A, and S4B). The low yield of potential 3D^pol^ interactors identified in these proteomics studies likely reflects the generally insoluble nature of ectopically expressed 3D^pol^ [38] and the low level of expression of HA-tagged 3D^pol^ that we were able to achieve in Huh-7.5 cells (S3A Fig).

Independent co-immunoprecipitation experiments confirmed the association of both HSPA8 and BAG2 with HA-tagged 3D^pol^-R468 expressed in Huh-7.5 cells (S5A Fig), validating these proteomics results. Similar co-immunoprecipitation assays confirmed that heterogenous nuclear ribonuclear protein H1 (hnRNPH1), and the DNA repair protein X-ray repair cross-complementing protein 6 (XRCC6), co-immunoprecipitate with HA-tagged 3D^pol^-R468 (S5B Fig). To determine whether BAG2 or either of these other candidate interactors are required for HAV replication, as HSPA8 is [11,18], we transfected Huh-7.5 cells with small interfering RNAs (siRNA) specific for each, and assessed the impact on replication of a nanoluciferase-expressing reporter virus, 18f-NLuc [39]. Overall, depletion of any of these 3D^pol^-interactors had relatively little impact on nanoluciferase expression as a measure of the replication of the reporter virus (S5C Fig). Nonetheless, BAG2 depletion consistently reduced nanoluciferase expression by approximately 20% at both early and late time points following infection (12, 24, and 48 hrs postinfection), whereas hnRNPH1 and XRCC6 depletion impaired replication only at 48 hrs (S5C and S5D Fig).

### Host species-specific effects of the 3D^pol^ R468K mutation on 3D^pol^-HSP70 complex formation

Since efficient HAV replication has been shown previously to require HSPA8 and the HSP70-HSP90 chaperone pathway in which BAG2 plays an important role [11,18–20], we focused on host-species specific differences in the interaction of the 3D^pol^ polymerase with HSPA8 and BAG2. Lysates from human Huh-7.5 and murine AML12 cells transfected with vectors expressing HA-tagged 3D^pol^-R468 or 3D^pol^-K468 were immunoprecipitated with anti-HA antibody, and the precipitates assayed for HSPA8 by immunoblotting. Quantitation of the results from 3 independent experiments revealed that significantly more HSPA8 was co-immunoprecipitated with the mouse-adapted 3D^pol^-K468 polymerase than with the wild-type 3D^pol^-R468 polymerase in lysates from the murine cells (Fig 6E and 6F). By contrast, more HSPA8 co-immunoprecipitated with the wild-type 3D^pol^-R468 polymerase than 3D^pol^-K468 in lysates from human cells (Fig 6E and 6F). Similar reciprocal quantitative differences were evident in the co-immunoprecipitation of BAG2 with HA-tagged 3D^pol^-R468 or 3D^pol^-K468 in expressed in Huh-7.5 versus AML12 cells (Fig 6E and 6G). These results provide strong evidence that the mouse adaptive R468K mutation promotes the association of 3D^pol^ with HSPA8 and BAG2 in cells of murine origin, likely facilitating HSP70-dependent post-translational maturation and acquisition of function by the polymerase.

## Discussion

Human HAV (*Hepatovirus ahepa* or *Hepatovirus A*) is one of at least 9 distinct hepatovirus species that infect a wide variety of mammalian host species, including seals, woodchucks, multiple species of rodents, and bats [40–43]. Comparative phylogenetic analyses of these hepatovirus species and their mammalian hosts are indicative of multiple host species jumps in the distant past [40,44]. Ancestral state reconstructions suggest a rodent origin for HAV [40], making it of particular interest to examine potential barriers to replication of this human virus in murine cells. Previous studies suggest that the major restriction to replication in mice rests in the inability of the virus to disrupt type I interferon responses, as the virus replicates well in mice deficient in the type I interferon receptor [4,7]. Some murine cell lines are also permissive for HAV infection [17,45], indicating that there is no substantial restriction to viral entry into mouse cells. This is consistent with studies indicating that endosomal gangliosides, which are broadly conserved among mammalian species, function as an essential receptor for HAV [18]. In our early studies of HAV infection in immunoincompetent mice, only a single, conservative nonsynonymous mutation, the 3D^pol^-R468K mutation, was found in the virus after 4 initial passages in DKO mice (Fig 1B) [7]. This suggested that there is little need for adaptive changes in the replication machinery of the virus in mouse cells. The studies we report here show that this early assumption was incorrect, and that changes in the thumb domain of the 3D^pol^ polymerase are required for efficient replication in mice.

Two independent lines of evidence support the conclusion that the 3D^pol^ mutations we have identified are mouse-adaptive. First, the R468K mutation was identified in three independent viral lineages recovered from mice infected either by intravenous inoculation of wild-type HAV (mp2 and mp4 virus) [7] or intrahepatic injection of wild-type, *in vitro* transcribed RNA (mRYp2 and mRXp2 viruses) (Fig 2A and Table 1). We cannot rule out the possibility that mutations in other viral proteins might promote replication in mice, but sequencing of the nearly complete genomes of two independently mouse-adapted viruses (mp4 and mRXp2) revealed adaptive mutations (R468K or D473G) only in 3D^pol^ (Fig 2A). Moreover, continued serial passage of the mp4 virus for almost 3 months in highly immunoincompetent *Mavs*^*-/-*^ mice resulted in no additional adaptive mutations elsewhere in the genome (Fig 1). More direct evidence for the mouse adaptive nature of the 3D^pol^ mutations comes from the reverse molecular genetics experiments that confirmed viral RNAs with the 3D^pol^-R468K or 3D^pol^-D473G mutations are more likely to initiate infection and replicate to higher levels than wild-type RNA when injected into the livers of either *Ifnar1*^*-/-*^ or *Mavs*^*-/-*^ mice (Fig 2B-2E). Overall, in our combined experiments, we were able to document infection in only 4 of 30 (13%) *Mavs*^*-/-*^ or *Ifnar1*^*-/-*^ mice injected with wild-type HAV RNA, versus 18 of 20 (90%) mice inoculated with 3D^pol^-K468 RNA. Despite this evidence that 3D^pol^-K468 enhances the capacity of HAV to replicate in mice, *in vitro* competition experiments with a cell culture-adapted virus demonstrated that virus containing this mutation is rapidly out-replicated by virus with the wild-type 3D^pol^-R468 in human cells (Fig 3B). Thus, the 3D^pol^-R468K mutation has a host species-specific effect on viral replication.

Relatively little is known about the 3D^pol^ protein of HAV. Its sequence is highly conserved, and residues E461, R468, and D473 are generally invariant in primate *Hepatovirus A* virus sequences. By contrast, each of these residues is different in the only *Hepatovirus A* virus identified thus far in a naturally-infected non-primate host, an alpaca in Bolivia [46]. The 3D^pol^ protein of the alpaca virus (GenBank OR452340.1) shares 89.2% amino acid identity and 96.5% similarity with the human HM175 virus polymerase, but has substitutions at each of the residues identified as sites of mouse adaptation in the human virus: E461D (as found in the mouse-adapted mRY lineage, Table 1), R468T, and D473N. It seems likely that these substitutions in the thumb domain of the polymerase may be alpaca-specific adaptations.

The close positioning of the species-specific 3D^pol^ mutations on the solvent-exposed surface of the thumb domain, as modeled with high confidence by AlphaFold 3 (Fig 5), suggests they promote an interaction with the murine ortholog of a previously unrecognized hepatovirus host factor that interacts with the polymerase. An alternate possibility is that the mutations reduce the affinity of an unknown murine restriction factor that binds the polymerase. Species-specific host factors that interact with and promote the activity of viral RNA polymerases are not unknown. A key example is the acidic leucine-rich nuclear phosphoprotein 32 family member A (ANP32A), that interacts with the PB2 subunit of the influenza A virus polymerase to regulate replicase activity in a host cell-species specific manner [2]. However, proteomics studies designed to identify such a factor for hepatoviruses yielded relatively few 3D^pol^-interacting candidates (Fig 6C). The small number of these candidates, and the relatively low level of specific enrichment of any of them in 3D^pol^ immunoprecipitates (Fig 6A and 6B), reflects the low level of expression of soluble HA-tagged 3D^pol^ that we were able to achieve in Huh-7.5 cells (S3A Fig). It is notable that previous efforts to express the polymerase in mammalian cells found it to be “virtually completely insoluble” [38]. Other evidence suggests it is subject to ubiquitin-dependent degradation by the 26S proteasome [47]. Unlike other picornaviral polymerases, it has never been expressed and purified in a catalytically-active form despite considerable effort [38].

HSPA8, a well-defined host factor essential for HAV infection, and its co-chaperone BAG2 were among the few candidate 3D^pol^ interactors identified by proteomics (Fig 6C). HSPA8 is one of eleven 70 kDa heat shock proteins (HSP70) expressed in Huh-7.5 cells [11,48]. It binds to and mediates the ATP hydrolysis-dependent folding of nascent client proteins in an early step in the HSP70/HSP90 chaperone pathway, prior to the transfer of client proteins to HSP90 [49,50]. BAG2, a nucleotide exchange factor, interacts directly as a dimer with substrate-bound HSPA8 to promote the release of ADP, accelerating this process by triggering HSPA8 release from its client protein, thereby enabling the transfer of client proteins to HSP90 [19,20]. HSPA8, DnaJ homolog subfamily A member 1 (DNAJA1, an additional HSP70 co-chaperone), and stress-induced phosphoprotein 1 (STIP1, which mediates the association of HSPA8 with HSP90), were all identified as high confidence HAV host factors in previous genome-wide CRISPR screens [18]. JG98 and VER155008, small molecule chemical inhibitors of HSP70 chaperone activity also demonstrate substantial antiviral activity against HAV, whereas HSP90 inhibitors potently block HAV replication in cell culture and infection in *Ifnar1*^*-/-*^ mice [11]. Multiple studies have shown the HSP70/HSP90 chaperone pathway is required for the proper folding and assembly of picornaviral capsids [51,52], and we have shown previously that HSP90 associates with the major capsid proteins of HAV in infected cells [11]. However, the replication of subgenomic HAV RNA replicons is also highly dependent upon the HSP70/HSP90 chaperone pathway, whereas this is not the case for poliovirus, rhinovirus or aphthovirus replicons [11,51,52].

Although the specific nonstructural HAV protein(s) requiring HSP70/HSP90 chaperone activity for functional maturation have not been identified, previous studies have implicated 3D^pol^ as a likely candidate [11]. The major viral protease, 3C^pro^, functions independently of HSP90 activity both in *cis* and in *trans*, and neither it nor the HAV 2B or 2C proteins were found to associate with HSP90 in proteomics studies of infected cells [11]. By contrast, 3D^pol^ expression was so low in such cells that no 3D^pol^-derived peptides were identified by mass spectrometry in cell lysates [11]. A requirement for chaperone activity would be consistent with the generally insoluble nature of ectopically-expressed 3D^pol^ and possible ubiquitin-dependent degradation of 3D^pol^ reported previously [38,47]. In light of these previous studies, the co-immunoprecipitation of HSPA8 and its co-chaperone regulator, BAG2, with ectopically expressed 3D^pol^ suggests 3D^pol^ is a client for the HSP70/HSP90 pathway, requiring chaperone activity to fold properly and acquire function. The reciprocal co-immunoprecipitation assays that show wild-type R468 polymerase co-immunoprecipitates more robustly with HSPA8-BAG2 in human cells, and the mouse-adapted K468 mutant more robustly with HSPA8-BAG2 in murine cells (Fig 6E-6G), thus provide a plausible mechanism for the mouse-adaptive nature of the R468K mutation. By facilitating the interaction of 3D^pol^ with HSPA8, BAG2 or other members of the HSP70/HSP90 pathway in murine cells, the R468K mutation likely promotes post-translational acquisition of polymerase function.

Our data are consistent with R468K and other mouse-adaptive mutations having a host-species specific effect on 3D^pol^-catalyzed RNA synthesis. However, like other picornaviral proteins, HAV 3D^pol^ is likely to be multifunctional. Although never demonstrated for HAV, other picornaviral polymerases are required for uridylation of VPg (3B), the protein primer for viral RNA synthesis [53]. The HAV 3CD protease-polymerase processing intermediate accumulates to detectable levels and, like 3C^pro^, is proteolytically active [12,38]. It may contribute to disruption of interferon responses in infected cells by cleaving the innate immune signaling adapter protein, TRIF [12]. Future studies should explore how mouse-adaptive 3D^pol^ mutations might influence these alternative 3D^pol^ functions, although it seems likely that proper folding and functional maturation of the polymerase mediated by HSP70/HSP90 would be essential for each.

Although the R468K mutation in 3D^pol^ substantially enhances the fitness of wild-type HAV RNA for replication in the murine liver, it was not able to rescue replication of cell culture-adapted p16 virus RNA inoculated into the livers of *Ifnar1*^*-/-*^ mice (Fig 4B and 4C). This provides interesting insight into the epistatic nature of the attenuating mutations in the genome of p16 that enhance fitness of the virus in cell culture, yet act in a dominant fashion to negate the phenotype of the 3D^pol^-R468K mutation in mice [28,36]. While the specific mutation(s) that attenuate the replication and virulence of p16 virus *in vivo* have yet to be mapped, previous studies suggest they are likely to involve amino acid substitutions in the 2B and/or 2C proteins that promote replication of the virus in cultured cells (Fig 4A) [28,34,35]. Recent studies published by others have shown that cell-culture adaptive mutations identified previously in 2B and 2C, when introduced into the genome of our mouse-adapted mp4 virus, both enhance the capacity of mp4 to replicate in cultured human hepatoma cells and ablate its ability to replicate in mice [35]. These recent findings complement our results and together with them collectively demonstrate the complex polygenic nature of HAV fitness. They highlight the varied strengths of the fitness advantage conferred by adaptation to a different host species, versus adaptation to the intracellular environment of cultured cells outside the liver. Although a molecular explanation for the apparent strict hepatotropism of HAV remains lacking, these data confirm its overarching importance to the biology of the virus.

Our identification of mouse adaptive mutations in 3D^pol^ has made possible direct reverse molecular genetics studies of HAV replication, virulence, and attenuation in mice. This represents a major technical advance, as such experiments have not been possible previously except in nonhuman primates. Although our data are consistent with a greater affinity of the K468 mutant for HSPA8 and/or BAG2 in murine cells (Fig 6E-6G), more refined and quantitative biophysical measurements, such as those that could be made by surface plasmon resonance instruments, will be required to definitively determine how mutations in the thumb domain of 3D^pol^ alter its affinity for individual components of the HSP70/HSP90 chaperone pathway in mice. Previous studies provide strong support for the critical role played by the chaperone pathway *in vivo*, including in infected mice [11], whereas our experiments have been limited by the need to express the polymerase ectopically in order to assess how mutations within it may affect its interactions with host proteins. Ideally, these interactions should be studied in infected cells. Such studies are likely to be exceptionally difficult technically, however, given the very low levels of 3D^pol^ expressed in infected cells and the generally insoluble nature of ectopically expressed 3D^pol^ [11,38].

## Materials and methods

### Ethics statement

Animals were bred and housed in a facility managed by the Division of Comparative Medicine at the University of North Carolina at Chapel Hill in accordance with the policies and guidelines of the Institutional Animal Care and Use Committee (IACUC) of the University of North Carolina at Chapel Hill. All animal procedures were approved by the UNC IACUC (IACUC Protocol #24-139.0).

### Cells

Huh-7.5 cells [54], a gift from Charles Rice of Rockefeller University, were maintained in DMEM with 3–10% fetal bovine serum and 1% penicillin/streptomycin. AML12 cells, which are transgenic mouse hepatocytes expressing transforming growth factor alpha (TGFα) [55], were obtained from the American Type Culture Collection, and propagated in DMEM/F-12 medium supplemented with 10% FBS, 1X insulin-transferrin-selenium (Gibco), 40 ng/ml dexamethasone, and 2 mM glutamine at 37°C in a 5% CO2 atmosphere, as described previously [17]. Cells tested negative for mycoplasma by PCR assay (LookOut Mycoplasma PCR Detection Kit, Sigma).

### Virus

All experiments utilized the HM175 strain of HAV. The HM175/mp4 virus inoculum was generated from lysate of mouse liver following 4 passages of primate-derived virus in *Ifnar1*^*-/-*^*Ifngr1*^*-/-*^ (DKO) mice, as described [7]. Additional liver- and fecal-derived viral inocula generated following viral passage or intrahepatic inoculation of in vitro transcribed RNA were prepared as described previously [7]. The cell-culture-adapted HAV variant, HM175/p16 (GenBank KP879217.1) [28], and HM175/18f nanoluciferase (NLuc) reporter virus, 18f-NLuc [18], were recovered from molecular clones and have been described previously.

### Plasmids

pHAVwt.2 (wt) is an infectious molecular clone of wild-type HM175 strain HAV (GenBank M14707.1) [23]; pHAV-3D^pol^/K468 and pHAV-3D^pol^/G473 were generated from pHAVwt.2 by PCR mutagenesis. pHAV/p16.2 is an infectious molecular clone of cell culture adapted HM175/p16 virus (GenBank KP879217.1) [28]; p16-3D^pol^/K468 and p16-3D^pol^*/G*473 were generated from pHAV/p16.2 by PCR mutagenesis. The expression vectors pHA-3D^pol^-R468 and pHA-3D^pol^-K468 contain sequence encoding N-terminally HA-tagged 3D^pol^ polymerase from HM175/18f virus (GenBank KP879216.1), without and with the R468K substitution, under control of the CMV promoter [14]. The complete nucleotide sequences of these plasmids were confirmed by next-generation DNA sequencing.

### Antibodies

Antibodies used in this study included: rabbit monoclonal anti-HA-Tag, C29F4 (Cell Signaling Technology, #3724); recombinant rabbit anti-Hsc70 (HSPA8) (Proteintech, #81719–2-RR); rabbit anti-BAG2 (Proteintech, #29820–1-AP); rabbit anti-hnRNPH1 (Proteintech, #14774–1-AP); rabbit anti-RACK1 (Proteintech, 27592–1-AP); rabbit anti-Ku70 (XRCC6), a gift from Dale Ramsden, The University of North Carolina at Chapel Hill; recombinant rabbit anti-ANT1/2 (SLC25A5) (Proteintech, #83104.1); rabbit monoclonal anti-GAPDH (14C10) (Cell Signaling Technologies, #2118); rabbit anti-β-actin (Cell Signaling Technologies, #4967); and, IRDye 800CW donkey anti-rabbit IgG (LICOR, #926–32213).

### Chemical reagents

The S-isomer of the antiviral compound RG7834 was purchased from MedChemExpress (#HY-117650A), and used to assess HAV replication at a concentration of 200nM.

### Animal studies

All mice were bred and housed in a facility managed by the Division of Comparative Medicine at the University of North Carolina at Chapel Hill in accordance with the policies and guidelines of the Institutional Animal Care and Use Committee (IACUC) of the University of North Carolina at Chapel Hill. All animal procedures were approved by the IACUC (IACUC Protocol #24-139.0). *Ifnar1*^*-/-*^ mice [56] had been backcrossed to B6-derivative mice at the Scripps Research Institute (La Jolla, CA) and at the University of North Carolina at Chapel Hill; *Mavs*^*-/-*^ mice were purchased from the Jackson Laboratory (JAX#008634). HAV infections were carried out in cohorts of 6–10 wks old male mice by intravenous injection of 2x10^5^ - 2x10^6^ genome equivalents (GE) of mouse-passaged virus using inocula prepared from lysates of mouse liver or fecal extracts, as described previously [7], or by direct intrahepatic inoculation of 0.1-50 μg *in vitro*-transcribed HAV RNA. Fecal pellets, collected from mice housed in individual cages, and liver tissue harvested at necropsy and stored in RNAlater (ThermoFisher Scientific), were assayed for HAV RNA by RT-qPCR. Infections in *Ifnar1*^*-/-*^ mice were monitored prior to necropsy by following serum ALT activity in serum samples collected from the tail vein of animals at intervals. Since there is no liver pathology associated with HAV infection in *Mavs*^*-/-*^ mice (no ALT elevation) [7], infections in *Mavs*^*-/-*^ mice were monitored by following the fecal shedding of virus detected by RT-PCR. Experiments designed to compare replication capacity or pathogenicity of different viruses or in vitro-transcribed RNAs utilized only male animals, as recent studies have shown that both the replication and pathogenicity of HAV is significantly greater in adult male mice than in female mice, making male animals a more sensitive test system for assessing potential differences [22].

### Serum Alanine Aminotransferase (ALT) assay

Mouse sera (2.5 μl) were diluted 1:2 in PBS and assayed for ALT activity using the Alanine Aminotransferase Activity Assay kit (Elabscience, #E-BC-K235-M).

### Quantitative RT-PCR assays

Total RNA was extracted from cultured cells using the RNeasy Kit (Qiagen, #74104), and from mouse liver tissue using TRIzol reagent (ThermoFisher, #15596026). Fecal RNA was isolated using the QIAamp Viral RNA Isolation Kit (Qiagen, #52906). cDNA synthesis was carried out using the SuperScript III First-Strand Synthesis System (ThermoFisher, #18080051). Primers targeting the 5’ untranslated region of the HAV genome were used to quantify HAV RNA genome equivalents (GE) in a SYBR Green Real-Time qPCR assay (BioRad, #1725121) against a synthetic RNA standard, as described previously [7].

### HAV genome sequencing

RNA was extracted from the livers of infected mice using TRIzol reagent (ThermoFisher, #15596026), and reverse-transcribed into cDNA using the SurperScript III First-Strand Synthesis System (ThermoFisher, #18080051) and random hexamer primers (Invitrogen, #48190011). Overlapping cDNA segments were amplified using primer sets that span the nearly complete HAV genome (nucleotides 24–7478), or 3D^pol^ coding region, and subjected to automated Sanger DNA sequencing.

### RNA transcription, transfection, and viral competition

HAV RNA was transcribed *in vitro* from plasmid DNA using the MEGAscript T7 Transcription Kit (ThermoFisher, #AM1334) and purified using the RNeasy Mini Kit (Qiagen, #74104). RNA concentrations were determined using a NanoDrop Spectrophotometer. RNA transfections were carried out with the Trans-It mRNA Transfection Kit (Mirus Bio #MIR225) according to the manufacturer’s recommended procedure. Replication of virus was assessed in transfected cells by comparing viral RNA abundance determined by RT-PCR with similarly transfected cells treated with the potent antiviral RG7834 (200nM) [29].

To compare replication of virus with 3D^pol^ proteins containing R468 versus K468, we carried out a competition assay. The 3D^pol^-R468K mutation was introduced into pHM175/p16, and an approximately equal mixture of RNAs transcribed from R468 and K468-encoding plasmids transfected into human Huh-7.5 and murine AML12 cells in replicate T-75 flasks. The cell culture media were replaced with fresh medium at 24 hrs, and the cells split 1:3 at 7 and 10 days post-transfection. Cell pellets were collected on days when the cell cultures were split (days 7 and 10) and on day14 post-transfection when the experiment was terminated, and RNA extracted using the RNeasy Kit (Qiagen, #74104). cDNA synthesis was carried out using the SuperScript III First-Strand Synthesis System (ThermoFisher, #18080051) and random hexamer primers (Invitrogen, #48190011). HAV sequence was amplified by PCR using primers spanning a 487 bp segment of the 3D^pol^ and 3’UTR sequence: forward, 5’-AATAGTTTTCTCTCGAGATGTTCAG-3’; reverse, 5’-ATTTACTGATAAAAGAAATAAACAAACCTCAGAAATTTTAAG-3’. DNA amplicons were sequenced by Oxford Nanopore Technologies long read sequencing (Plasmidsaurus), and reads encoding 3D^pol^-R468 versus 3D^pol^-K468 enumerated. Changes in the proportion of reads encoding R468 versus K468 were assessed for significance using Fisher’s exact test and repeated measures ANOVA.

### 3D^pol^ co-immunoprecipitation assays

For immunoprecipitation of proteins associating with 3D^pol^, Huh-7.5 cells were transfected with pHA-3D^pol^-R468 or pHA-3D^pol^-K458 using FuGENE HD Transfection Reagent (Promega, #E2311) as described previously [57]. Cells were harvested by the addition of lysis buffer [150 mM KCl, 25 mM Tris-HCl pH 7.4, 5 mM EDTA, 1% Triton X-100, 5 mM DTT, cOmplete Protease Inhibitor Cocktail (Roche, #11697498001), 100 U/ml RNaseOUT Recombinant Ribonuclease Inhibitor (Invitrogen # 10777–019)]. Lysates were centrifuged, and supernatants incubated with anti-HA-Tag (Cell Signaling Technology, #3724) or control IgG at 4° C for 2 hrs, followed by the addition of magnetic Protein A/G Magnetic Beads (ThermoFisher, #88802). Following a 1 hr incubation at 4° C, beads were washed 4 times in lysis buffer, and proteins eluted in sample buffer for SDS-PAGE. For immunoblotting, the eluted proteins were mixed with 4 × Laemmli buffer, incubated at 95 °C for 5 min, then resolved in 4–15% or 4–20% gradient SDS–polyacrylamide pre-cast gels (BioRad, #4561086, #4560196). Proteins were then transferred to a polyvinylidene fluoride membranes by semi-dry transfer using the Transblot Turbo apparatus (BioRad). Membranes were blocked in Odyssey Blocking Buffer (LI-COR Biosciences) and probed with primary antibodies overnight, followed by washing in 0.05% Tween-20. The membranes were then incubated with a 1:10,000 dilution of donkey anti-rabbit secondary antibodies conjugated with an infrared label (IRDye 800, LI-COR Biosciences) for 1 h at room temperature. After additional washing with 0.05% Tween-20, protein bands were visualized using an Odyssey Infrared Imaging System (LI-COR Biosciences). Protein bands were quantified based on fluorescent intensity.

### Quantitative proteomics

Proteins precipitated with anti-HA-Tag antibody from lysates of cells transfected with pHA-3D^pol^-R468 or pHA-3D^pol^-K468, or mock transfected (3 biological replicates each) were eluted in SDS-PAGE sample buffer and run 1–2 cm into an SDS-PAGE gel. The gels were cut, destained, reduced and alkylated followed tryptic digestion. The peptides were extracted, desalted on home-made C18 stage-tips. The clean peptides were dissolved in 0.1% formic acid and analyzed on a Q-Exactive HF-X mass spectrometer coupled with an Easy nanoLC 1200 System (ThermoFisher Scientific, San Jose, CA). Peptides were loaded on to a nanoEase MZ HSS T3 Column (100Å, 1.8 μm, 75 μm x 250 mm, Waters). Analytical separation of the anti-HA-pulldown digests was achieved with a linear gradient of 5–30% buffer B over 29 min, and 30% to 45% buffer B over 6 min at a 250 nl/min flow rate, followed by a ramp to 100% B in 1 min and 14-min wash with 100% B, where buffer A was aqueous 0.1% formic acid, and buffer B was 80% acetonitrile and 0.1% formic acid. LC-MS was carried out in a data-dependent mode with a resolution of 60,000 at m/z 200 followed by high energy collision-activated dissociation-MS/MS of the top 15 most intense ions with a resolution of 15,000 at m/z 200. High energy collision-activated dissociation-MS/MS was used to dissociate peptides at a normalized collision energy of 27 eV in the presence of nitrogen bath gas atoms. There were two technical LC-MS replicates for each of three anti-HA precipitated samples.

Mass spectra were processed, and peptide identification carried out using MaxQuant software version 1.6.10.43 (Max Planck Institute, Germany). Protein database searches were carried out against the UniProt human protein sequence database (UP000005640). A false discovery rate (FDR) for both peptide-spectrum match (PSM) and protein assignment was set at 1%. Search parameters included up to two missed cleavages at Lys/Arg on the sequence, oxidation of methionine and protein N-terminal acetylation as a dynamic modification. Carbamidomethylation of cysteine residues was considered as a static modification. Peptide identifications were reported by filtering of reverse and contaminant entries and assigning to their leading razor protein. Intensity-based Absolute Quantitation (IBAQ) and Label-free Quantitation (LFQ) values were calculated with MaxQuant. Data processing and statistical analysis were done with Perseus version 1.6.10.50. Technical replicates were analyzed using two-sample t-test statistics with a p-value of 0.05 considered a significant fold-difference in protein abundance for proteins immunoprecipitated from different cell lysates.

### siRNA depletion of host proteins

For RNAi depletion of cellular proteins, cells were transfected with ON-TARGETplus SMARTpool siRNAs, each pool containing 4 individual siRNA molecules targeting human BAG2 (Entrez Gene 9532), human HNRNPH1 (3187), human RACK1 (10399), or human XRCC6 (2547), or ON-TARGETplus Non-targeting Control siRNA (Control #2, ‘siCtrl’) (Dharmacon). Transfections were carried out using the Lipofectamine RNAiMAX Transfection Reagent (ThermoFisher, #13778100) according to the manufacturer’s protocol.

### Statistical analysis

Statistical calculations were carried out with Prism 10 for Mac OS, version 10.6.1 (GraphPad). Specific types of statistical tests used included two-way t-tests, simple ANOVA, and repeated measures ANOVA depending on the specific experimental design, as described in the legends to the figures. In general, a p-value <0.05 was considered significance. Correction for multiple comparisons, if done, are described in the legends to each figure.

## Supporting information

S1 Table Nucleotide differences in plasmid and virus-derived RNA sequences. (XLSX)

S2 Table Intensity-based Absolute Quantitation (IBAQ) proteomics analysis. Intensity-based Absolute Quantitation (IBAQ) proteomics analysis of host cell proteins identified in anti-HA precipitates from HA-3D^pol^-R468 or HA-3D^pol^-K468 expressing Huh-7.5 cells, or mock transfected (control) cells.
(XLSX)

S3 Table Label-free Quantitation (LFQ) proteomics analysis. Label-free Quantitation (LFQ) proteomics analysis of host cell proteins identified in anti-HA precipitates from HA-3D^pol^-R468 or HA-3D^pol^-K468 expressing Huh-7.5 cells, or mock transfected (control) cells.
(XLSX)

S1 Fig Predicted secondary structure of the 3’UTR and upstream RNA sequence. Structure of the 3’UTR and upstream RNA encoding the carboxy terminus of 3D^pol^ in the wild-type HM175 virus genome (nts 7210–7500) predicted by RNA Structure v6.5 [58]. Bases at which nucleotide substitutions were identified in mouse-adapted viruses, 7331G→U (E461D), 7351G→A (R468K), and 7366A→G (D473G) are highlighted with the codon lightly shaded in pink, and the stop codon darkly shaded in red. Nucleotide substitutions that distinguish genotype V HAV recovered from a rhesus monkey (GenBank EU140838.1) from the human genotype 1a HM175 virus (M14707.1) are shown in boxes adjacent to bases in the HM175 sequence. Red boxes delineate co-variant nucleotide substitutions that support the predicted structures of stem-loops iii and iv. Stem-loops i and ii and base-pairing at the base of stem-loop iii are low confidence predictions. Free energy of the fold, ΔG = -66.4 kcal/mol.
(PDF)

S2 Fig AlphaFold 3 predicted 3D^pol^ structures. (A) Color-coded confidence of the predicted structure of wild-type HM175 HAV based on the per-residue local distance difference test score (pIDDT). (B) Superimposition of the HAV 3D^pol^ structure (green) on crystal structure of poliovirus 3D^pol^ (red) (Protein Data Bank accession 1rdr) [59]. (C) Superimposition of the predicted wild-type HAV 3D^pol^ structure (green) on the predicted structure of the mouse-adapted 3D^pol^-K468 mutant (salmon). Side chains are shown for residue 468. Root mean square deviation (RMSD) was 0.181. (D-F) Superimposition of parts of the predicted wild-type thumb domain structure (green) on predicted structures of the mouse-adapted 3D^pol^ mutants showing changes in side-chain length and orientation: (D) K468, (E) G473 (RMSD for the entire molecule = 0.231), and (F) D461 (RMSD = 0.227).
(PDF)

S3 Fig Immunoblots of HA-tagged 3D^pol^ expression products. (A) Anti-HA immunoblot of lysates (“Input”) from human Huh-7.5 cells transfected with vectors expressing 3D^pol^-R468, 3D^pol^-K468, or empty vector (“Control”) and related anti-HA immunoprecipitates (“IP”). Immunoprecipitated HA-3D^pol^ is shown in the red lined box. (B) Anti-HA immunoblot of triplicate anti-HA immunoprecipitate samples from Huh-7.5 cells subjected to LC-MS proteomics analysis.
(PDF)

S4 Fig Quantitative proteomics analysis of proteins co-immunoprecipitating with HA-3D^pol^-K468. (A,B) Volcano plots showing fold difference (FD) in abundance of cellular proteins co-immunoprecipitated from Huh-7.5 cells expressing HA-3D^pol^-K468 versus control cells (ctrl, mock transfected not expressing HA-3D^pol^), quantified by (A) LFQ or (B) IBAQ methods. Proteins with log_2_[FD]>0.5 and p < 0.05 are labeled in each panel. No protein reached this criterion by both methods. HSPA8 and BAG2 are highlighted in red and orange, respectively. (C) LFQ intensities of peptides derived from 3D^pol^, HSPA8, and BAG2 in anti-HA precipitates from control cells, and cells expressing HA-3D^pol^-R468, or HA-3D^pol^-K468. Data shown are means of replicate assays carried out on each of 3 independent immunoprecipitates. NaN, nonquantifiable. p-values by two sided t-test.
(PDF)

S5 Fig Validation of candidate 3D^pol^ interactor proteins. (A) Co-immunoprecipitation of (*top*) HSPA8 and (*bottom*) BAG2 with HA-3D^pol^-R468 expressed in Huh-7.5 cells, and vice versa. IP, immunoprecipitation; IB, immunoblot, with indicated antibodies. IgG, isotype control antibody. (B) Co-immunoprecipitation of HA-3D^pol^-R468 and HA-3D^pol^-K468 with candidate 3D^pol^ interactors other than HSPA8 that were sufficiently abundant to be quantifiable in the LFQ analysis (Fig 6C in main manuscript). Immunoblots show hnRNPH1, RACK1, SLC25A5, and XRCC6 in lysates (“Input”) and anti-HA immunoprecipitates from Huh-7.5 cells expressing HA-3D^pol^-R468, HA-3D^pol^-K468, or transfected with empty vector (Control). (C) Nanoluciferase (NLuc) expressed by the18f-NLuc reporter virus in Huh-7.5 cells following siRNA-mediated depletion of BAG2, hnRNPH1, or XRCC6. (*top*) Immunoblots showing target protein abundance in Huh-7.5 cells transfected with nontargeting siCtrl siRNA or target-specific siRNA. (*bottom*) NLuc expressed by 18f-NLuc virus over a 48 hr period following infection of cells transfected with siCtrl or target protein-specific siRNAs. *p < 0.05, **p < 0.01, ***p < 0.001 by two-sided unpaired t-test; n = 3 technical replicates. (D) Percent nanoluciferase expressed in cells depleted of candidate 3D^pol^ interactors shown in panel C 12, 24, and 48 hrs after 18f-NLuc infection, relative to siCtrl-transfected control cells. q-value by two-way repeated measure ANOVA with two-stage linear step-up procedure of Benjamini, Krieger and Yekutieli.
(PDF)
