# Supplementary figures and images for: Host species-specific mutations in the thumb domain of the 3Dpol polymerase are required for efficient replication of human hepatitis A virus in mice
Source: PLoS Pathog. 2026 May 11;22(5):e1014213. doi: 10.1371/journal.ppat.1014213 (PMC13175494; doi:10.1371/journal.ppat.1014213)

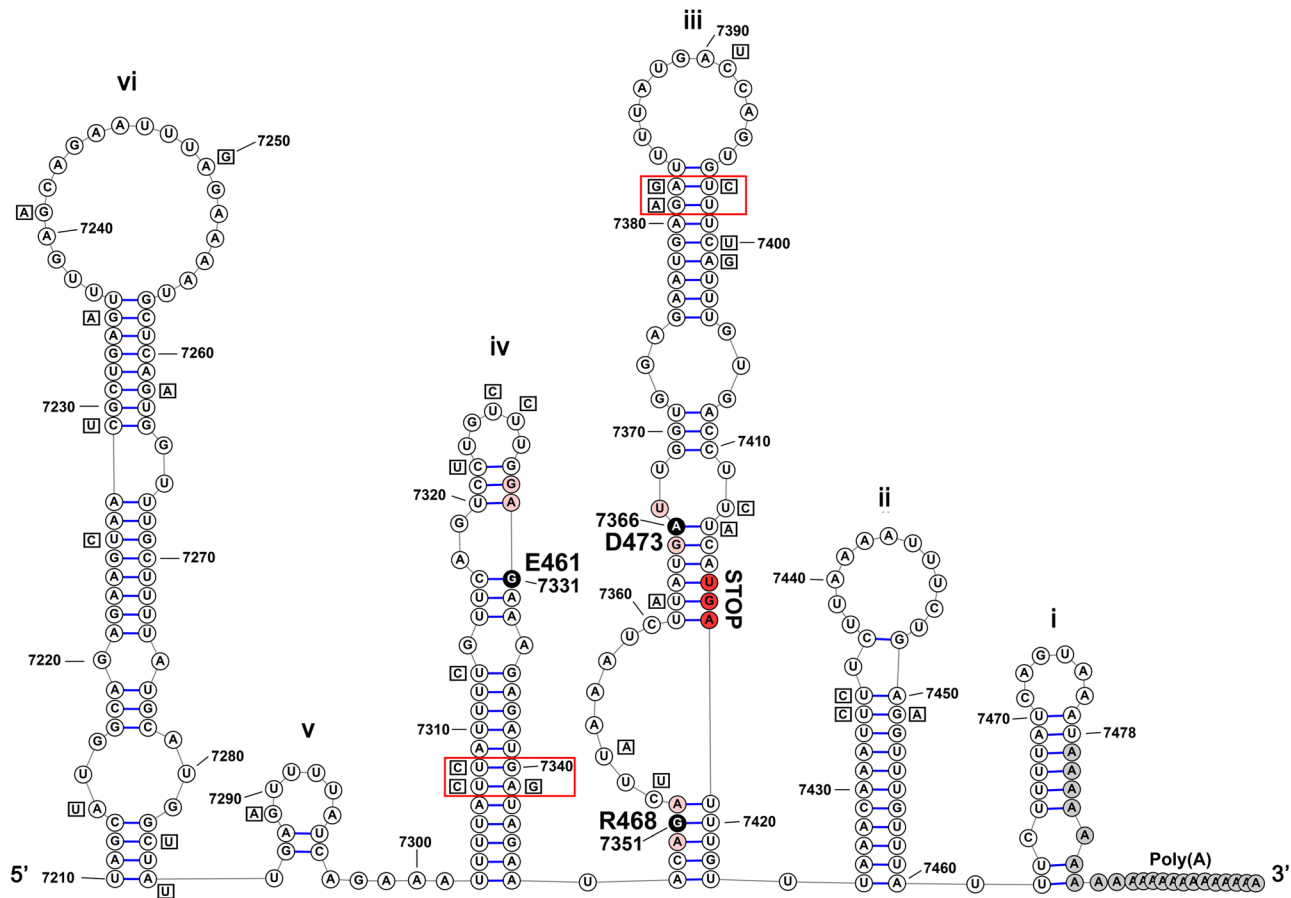

Supplement: S1 Fig — Structure of the 3’UTR and upstream RNA encoding the carboxy terminus of 3Dpol in the wild-type HM175 virus genome (nts 7210–7500) predicted by RNA Structure v6.5 [58]. Bases at which nucleotide substitutions were identified in mouse-adapted viruses, 7331G→U (E461D), 7351G→A (R468K), and 7366A→G (D473G) are highlighted with the codon lightly shaded in pink, and the stop codon darkly shaded in red. Nucleotide substitutions that distinguish genotype V HAV recovered from a rhesus monkey (GenBank EU140838.1) from the human genotype 1a HM175 virus (M14707.1) are shown in boxes adjacent to bases in the HM175 sequence. Red boxes delineate co-variant nucleotide substitutions that support the predicted structures of stem-loops iii and iv. Stem-loops i and ii and base-pairing at the base of stem-loop iii are low confidence predictions. Free energy of the fold, ΔG = -66.4 kcal/mol. (PDF) [file ppat.1014213.s004.pdf]

**A**

pIIDD  
>90 Very high  
90>70 Confident  
70>50 Low  
<50 Very low

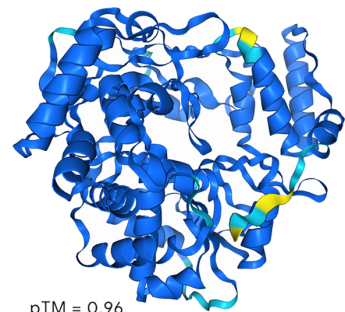

pTM = 0.96

**B****HAV****PV**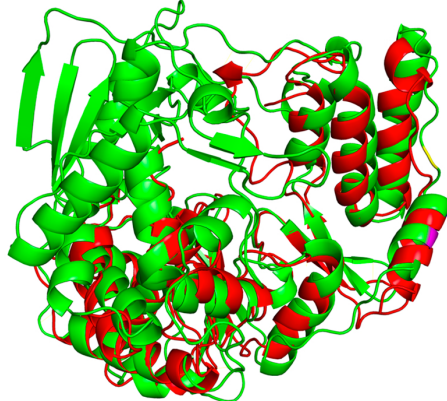**C****wt****K468**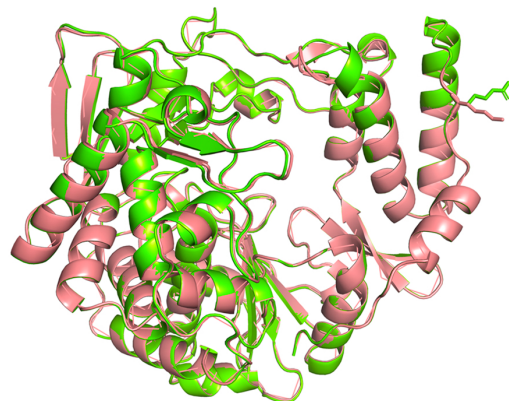**D****wt****K468**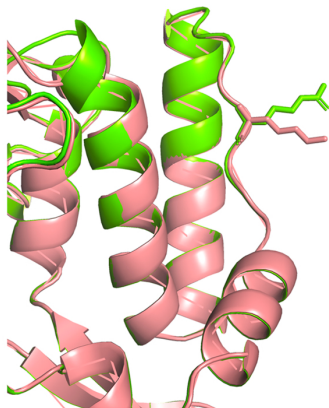**E****wt****G473**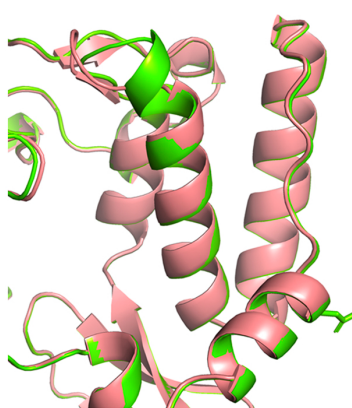**F****wt****D461**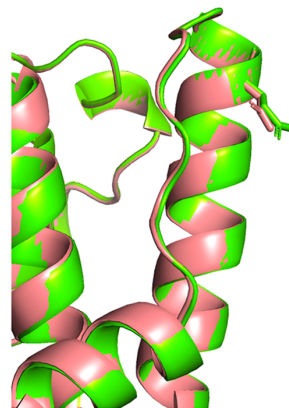

Supplement: S2 Fig — (A) Color-coded confidence of the predicted structure of wild-type HM175 HAV based on the per-residue local distance difference test score (pIDDT). (B) Superimposition of the HAV 3Dpol structure (green) on crystal structure of poliovirus 3Dpol (red) (Protein Data Bank accession 1rdr) [59]. (C) Superimposition of the predicted wild-type HAV 3Dpol structure (green) on the predicted structure of the mouse-adapted 3Dpol-K468 mutant (salmon). Side chains are shown for residue 468. Root mean square deviation (RMSD) was 0.181. (D-F) Superimposition of parts of the predicted wild-type thumb domain structure (green) on predicted structures of the mouse-adapted 3Dpol mutants showing changes in side-chain length and orientation: (D) K468, (E) G473 (RMSD for the entire molecule = 0.231), and (F) D461 (RMSD = 0.227). (PDF) [file ppat.1014213.s005.pdf]

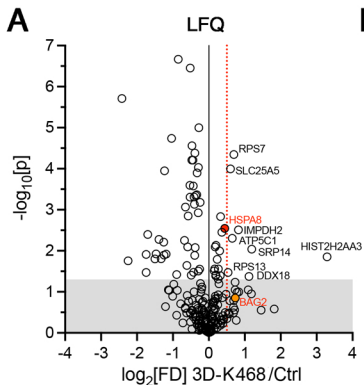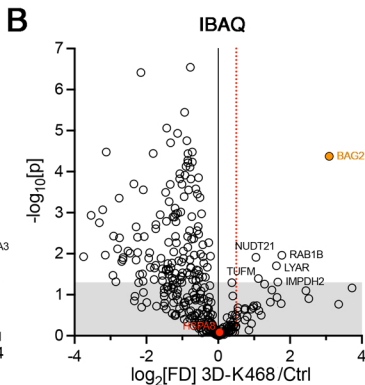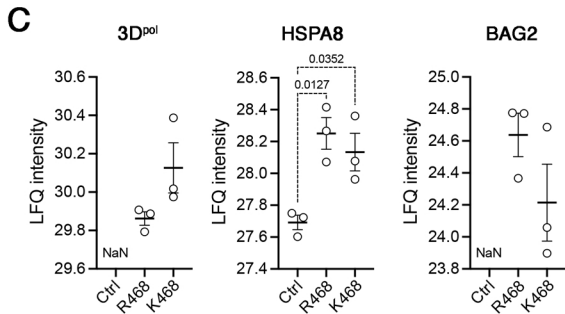

Supplement: S4 Fig — (A,B) Volcano plots showing fold difference (FD) in abundance of cellular proteins co-immunoprecipitated from Huh-7.5 cells expressing HA-3Dpol-K468 versus control cells (ctrl, mock transfected not expressing HA-3Dpol), quantified by (A) LFQ or (B) IBAQ methods. Proteins with log2[FD]>0.5 and p < 0.05 are labeled in each panel. No protein reached this criterion by both methods. HSPA8 and BAG2 are highlighted in red and orange, respectively. (C) LFQ intensities of peptides derived from 3Dpol, HSPA8, and BAG2 in anti-HA precipitates from control cells, and cells expressing HA-3Dpol-R468, or HA-3Dpol-K468. Data shown are means of replicate assays carried out on each of 3 independent immunoprecipitates. NaN, nonquantifiable. p-values by two sided t-test. (PDF) [file ppat.1014213.s007.pdf]

**A**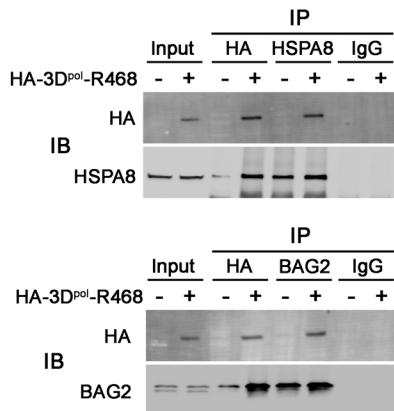**B**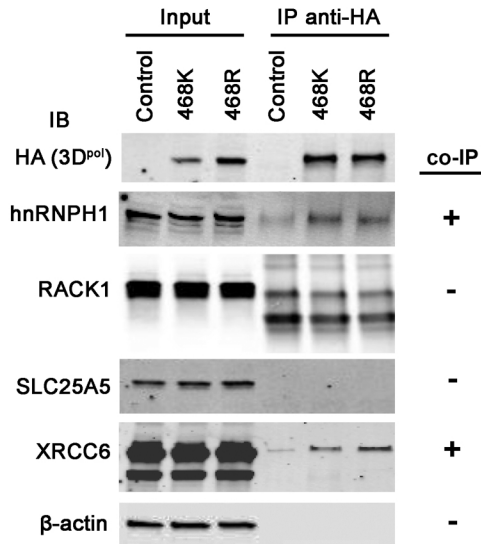**C**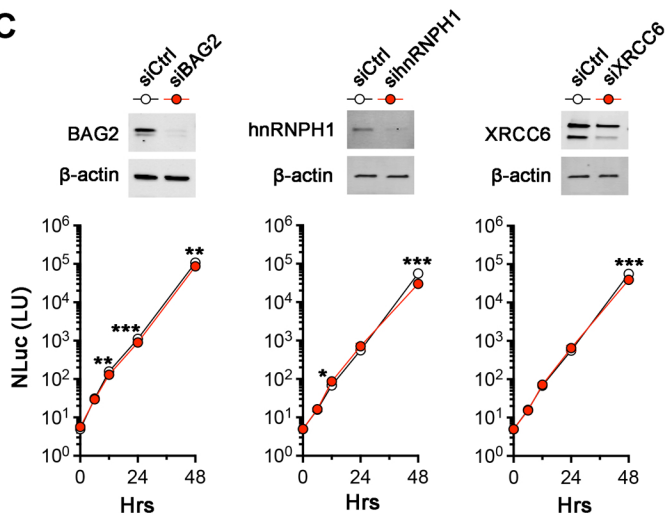**D**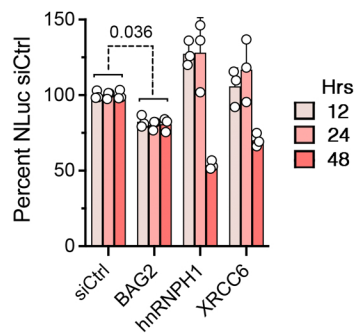

Supplement: S5 Fig — (A) Co-immunoprecipitation of (top) HSPA8 and (bottom) BAG2 with HA-3Dpol-R468 expressed in Huh-7.5 cells, and vice versa. IP, immunoprecipitation; IB, immunoblot, with indicated antibodies. IgG, isotype control antibody. (B) Co-immunoprecipitation of HA-3Dpol-R468 and HA-3Dpol-K468 with candidate 3Dpol interactors other than HSPA8 that were sufficiently abundant to be quantifiable in the LFQ analysis (Fig 6C in main manuscript). Immunoblots show hnRNPH1, RACK1, SLC25A5, and XRCC6 in lysates (“Input”) and anti-HA immunoprecipitates from Huh-7.5 cells expressing HA-3Dpol-R468, HA-3Dpol-K468, or transfected with empty vector (Control). (C) Nanoluciferase (NLuc) expressed by the18f-NLuc reporter virus in Huh-7.5 cells following siRNA-mediated depletion of BAG2, hnRNPH1, or XRCC6. (top) Immunoblots showing target protein abundance in Huh-7.5 cells transfected with nontargeting siCtrl siRNA or target-specific siRNA. (bottom) NLuc expressed by 18f-NLuc virus over a 48 hr period following infection of cells transfected with siCtrl or target protein-specific siRNAs. *p < 0.05, **p < 0.01, ***p < 0.001 by two-sided unpaired t-test; n = 3 technical replicates. (D) Percent nanoluciferase expressed in cells depleted of candidate 3Dpol interactors shown in panel C 12, 24, and 48 hrs after 18f-NLuc infection, relative to siCtrl-transfected control cells. q-value by two-way repeated measure ANOVA with two-stage linear step-up procedure of Benjamini, Krieger and Yekutieli. (PDF) [file ppat.1014213.s008.pdf]
