# Supplementary material for: Host species-specific mutations in the thumb domain of the 3Dpol polymerase are required for efficient replication of human hepatitis A virus in mice
Source: PLoS Pathog. 2026 May 11;22(5):e1014213. doi: 10.1371/journal.ppat.1014213 (PMC13175494; doi:10.1371/journal.ppat.1014213)
Supplement: S3 Fig — (A) Anti-HA immunoblot of lysates (“Input”) from human Huh-7.5 cells transfected with vectors expressing 3Dpol-R468, 3Dpol-K468, or empty vector (“Control”) and related anti-HA immunoprecipitates (“IP”). Immunoprecipitated HA-3Dpol is shown in the red lined box. (B) Anti-HA immunoblot of triplicate anti-HA immunoprecipitate samples from Huh-7.5 cells subjected to LC-MS proteomics analysis. (PDF) [file ppat.1014213.s006.pdf]

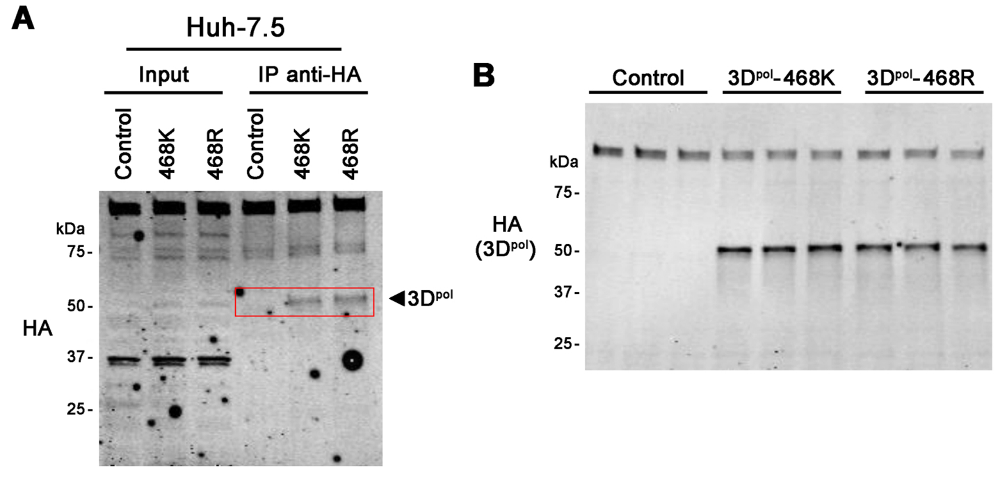
A
Huh-7.5
Input
IP anti-HA
Control
468K
468R
Control
468K
468R
kDa
75-
50-
37-
25-
HA
◀3Dpol
B
Control
3Dpol-468K
3Dpol-468R
kDa
75-
50-
37-
25-
HA
(3Dpol)
